# Epidural anesthesia for laparoscopic bariatric surgery: a case report

**DOI:** 10.1186/s40064-015-1153-x

**Published:** 2015-07-17

**Authors:** Wei-Chun Hung, Wei-Hung Chen, Yu-Hsuan Shih, Kuo-Chuan Hung

**Affiliations:** Department of Anesthesiology, E-DA Hospital, I-Shou University, 1, E-Da Road, Jiau-shu Tsuen, Yan-Chau Shiang, Kaohsiung, 824 Taiwan, ROC

**Keywords:** Epidural anesthesia, Laparoscopic bariatric surgery, Obesity

## Abstract

**Background:**

Rapid and uneventful postoperative recovery following general anesthesia in morbidly obese patients undergoing bariatric surgery may offer challenges to anesthesiologists. With improved surgical techniques and shorter pneumoperitoneum, regional anesthesia may be considered for this laparoscopic procedure in selected cases.

**Case description:**

The first patient was a 60-year-old male (body mass index: 39 kg/m^2^) who was scheduled for laparoscopic sleeve gastrectomy. The second patient was a 46-year-old female (body mass index: 47 kg/m^2^) who was scheduled for laparoscopic gastric bypass. After standard intraoperative monitoring was applied, epidural anesthesia was performed at thoracic level T9–T10. Surgical technique modification included insufflation of CO_2_ at a low flow rate and avoidance of orogastric tube use. During the procedure, both patients breathed spontaneously without difficulty. One hypotension episode occurred and was successfully treated with a 12-mg bolus of ephedrine in case 1. Shoulder pain occurred intraoperatively in case 2 and was successfully treated with a 50-μg bolus of fentanyl. Postoperatively, 2 mg epidural morphine was administered for postoperative analgesia. Both patients were satisfied with the anesthesia technique and was discharged uneventfully.

**Discussion and evaluation:**

This anesthetic technique may maintain pre-operative respiratory function, increase alertness, and reduce the use of rescue analgesics, which is crucial for optimal outcomes in morbidly obese patients. Conversion of epidural anesthesia to general anesthesia may be required if patients can not tolerate the laparoscopic procedure (e.g. intolerable shoulder pain) or the increased respiratory rate during pneumoperitoneum leading to difficulty in performing laparoscopic surgery. Further studies are needed to elucidate this issue.

**Conclusions:**

General anesthesia is widely used for laparoscopic bariatric surgery, but epidural anesthesia may be a viable alternative to general anesthesia in selected cases. Further prospective studies may be required to elucidate the relative advantages and disadvantages of epidural anesthesia in this surgical population.

## Background

Bariatric surgery, the only proven, effective, and durable treatment for morbidly obese patients (Brolin 2002), is commonly performed under general anesthesia with volatile anesthetics and neuromuscular blockade. However, general anesthesia with neuromuscular blockade increases upper airway collapse risk, pulmonary atelectasis, and postoperative pulmonary complications in this population (Pelosi et al. 1997). Acute respiratory failure, if occurs after bariatric surgery, may significantly increase in-hospital mortality (Masoomi et al. 2013). We report two case of successful laparoscopic bariatric surgery under epidural anesthesia. With improved surgical techniques and shorter pneumoperitoneum, regional anesthesia may be considered as be a viable alternative to general anesthesia in selected cases.

## Case report

The first patient was a 60-year-old male (weight: 115 kg; height: 172 cm; body mass index: 39 kg/m^2^) who was scheduled for laparoscopic sleeve gastrectomy. His past history included tobacco-related chronic obstructive pulmonary disease (COPD) and asthma. Preoperative pulmonary function tests revealed severe obstructive impairment, with forced vital capacity (FVC) 1.29 L, one-second forced expiratory volume (FEV1) 0.76 L, and FEV1/FVC 59%. The second patient was a 46-year-old female (weight: 115 kg; height: 157 cm; body mass index of 47 kg/m^2^) who was scheduled for laparoscopic gastric bypass. Her past history included diabetes mellitus, hypertension, and obstructive sleep apnea. Both patients had an ASA physical status 3 and physical examination was unremarkable. The airway, assessed with the patients in the sitting position, was judged to be Mallampati class I–II.

Both patients were scheduled for laparoscopic bariatric surgery because of failing conservative weight loss measures. The operative criteria were based on the Asia–Pacific guidelines for bariatric surgery, i.e. body mass index ≧32 kg/m^2^ in the presence of comorbidities and body mass index ≧37 kg/m^2^ with or without comorbidities. At the preoperative visit, both patients opted for epidural anesthesia after a detailed explanation regarding postoperative pulmonary complication risks. Epidural anesthesia and the possibility of intraoperative conversion to general anesthesia were explained.

Standard intraoperative monitoring included electrocardiography, noninvasive blood pressure, and pulse oximetry. After obtaining baseline vital signs, oxygen (6 L/min) was commenced through a face mask. Both patients received ketorolac 30 mg and meperidine 30 mg as an analgesic premedication to relieve procedural discomfort. Before epidural anesthesia, they received Ringer’s lactate solution (500 ml). With both patients at the right lateral decubitus position, an 18-gauge Tuohy-Huber needle was introduced at the T9/T10 intervertebral space using the paramedian approach under full aseptic precautions. After “loss of resistance” to air was identified, a 20-gauge epidural catheter was threaded into place. The tip of the catheter was advanced 4–6 cm cephalad beyond the needle tip. After the catheter was taped to the patient’s back, they were turned to the supine position. Thigh-length antiembolic stockings and sequential pneumatic compression devices were placed on both lower extremities for venous thromboembolism prophylaxis. Urine catheter was not inserted.

After intravascular/intrathecal injection was excluded by injecting a 3-ml test dose of 2% lidocaine and epinephrine (1: 200,000), an intermittent bolus of 0.5% bupivacaine and 5 µg/ml fentanyl was injected to obtain segmental sensory block (pinprick) till the fourth thoracic dermatomes. The laparoscopic bariatric surgery was performed by an experienced surgeon, who performed 250–300 cases of laparoscopic bariatric surgery per year. Pneumoperitoneum was established with carbon dioxide at an intra-abdominal pressure of 13 mmHg. To avoid vasovagal reflex and shoulder pain, the carbon dioxide was insufflated at a low flow rate (2 L/min) (Yuksek et al. 2008). Then both patients was placed in reverse Trendelenburg position at 30°–45° angle. Calibrating orogastric tube was not used for gastric pouch formation. Laparoscopic bariatric surgery was performed according to the technique described before (Zachariah et al. 2013**)**. No other intraoperative sedatives were administered.

In case 1, one episode of hypotension (systolic blood pressure <90 mmHg) occurred after epidural anesthesia, and a 12-mg ephedrine bolus was intravenously administered. Afterward, his hemodynamic changes were minimal. The patient was alert and breathed spontaneously without difficulty. Intraoperatively, no shoulder pain or other discomfort was described. In case 2, shoulder pain occurred during penumoperitoneum and fentanyl 50 μg was given intravenously to relieve the shoulder pain. No episode of hypotension or desaturation was observed during the procedure. The surgeon considered the abdominal relaxation was acceptable in both patients. However, increased respiratory rate in case 1 caused a slight disturbance during laparoscopic exploration. The procedure was completed without complications (operative time: 49 min). In case 2, the laparoscopic procedure was completed without difficulty (operative time: 56 min).

At the end of the operation, 2 mg morphine plus 14 ml normal saline was administered via the epidural catheter for postoperative analgesia in both patients. Then, the epidural catheter was removed. Both patients were able to move unassisted from the operating table to their bed after the operation. Postoperative recovery was uneventful. They did not experience nausea, vomiting, itching, urine retention, or respiratory depression. Rescue analgesics for pain were not required within 24 h postoperatively. Both patients were satisfactory with the anesthetic technique at the postoperative visit, and were discharged on postoperative day three.

## Discussion

Obese patients often have poor pulmonary functional residual capacity and other spirometric parameters. As general anesthesia may cause increased intrapulmonary shunting and atelectasis, postoperative pulmonary complications are common in morbidly obese patients after laparoscopic bariatric surgery (Reinius et al. 2009). Respiratory failure in morbidly obese patients is associated with greater in-hospital mortality after bariatric surgery (Masoomi et al. 2013). Patients under regional anesthesia usually develop fewer pulmonary complications than those under general anesthesia (Pedersen et al. 1992); this is why we chose epidural anesthesia for this procedure.

Carbon dioxide pneumoperitoneum may adversely affect intraoperative pulmonary mechanics (Nguyen et al. 2004). Therefore, our patient was at risk of intraoperative pulmonary distress. In a previous small feasibility study (n = 23), Symeonidis et al. (2013), found that obese patients (mean body mass index: 36 kg/m^2^) tolerate laparoscopic ventral hernia repair well without intraoperative pulmonary complications. Also, there are several reported cases of uneventful laparoscopic cholecystectomies that used regional anesthesia in patients with severe pulmonary disease (Kim et al. 2007; van Zundert et al. 2006; Pursnani et al. 1998). Pneumoperitoneum-induced respiratory distress did not occur in our patients. To maintain the reverse Trendelenburg position at 30°–45° angle during procedure and short duration of pneumoperitoneum may have also contributed to this.

There are several advantages to using regional anesthesia for laparoscopic bariatric surgery in both cases. First, preoperative pulmonary function parameters may be maintained throughout the surgery (Kim et al. 2007; van Zundert et al. 2006; Pursnani et al. 1998). In contrast, pulmonary function parameters may not return to preoperative levels until the seventh postoperative day in patients receiving general anesthesia (Nguyen et al. 2001). Secondly, both the laparoscopic procedure (Nguyen et al. 2002) and invasive tracheal intubation (Carron et al. 2012) may induce significant systemic stress response, which can have a potential detrimental effects in high co-morbidity obese patients. Using epidural anesthesia for our patient helped avoid airway intervention and attenuated sympathetic responses caused by the surgical insult (Aono et al. 1998); this likely improved his postoperative outcomes and accelerated baseline function return. Finally, morbid obesity is generally concluded to be a risk factor for the development of perioperative deep venous thrombosis and pulmonary embolism (Wu and Barba 2000). The risk of deep venous thrombosis and pulmonary embolism is lower with epidural anesthesia than with general anesthesia (Rodgers et al. 2000).

Shoulder pain, caused by diaphragmatic irritation by insufflation gas, is common during laparoscopic surgery under regional anesthesia. A fentanyl bolus dose may relieve the shoulder pain; however, conversion of regional to general anesthesia may be necessary in some cases. In a prospective study (n = 180), the authors reported that the frequency of anesthetic conversions due to intolerable shoulder pain during laparoscopic cholecystectomy is 4.4% (Bessa et al. 2012). Hypotension caused by neuraxial blockade is also common during laparoscopic surgeries (Lee et al. 2010), but it can be easily controlled with ephedrine, as in our case.

In our patients, 2 mg epidural morphine was administered for postoperative analgesia, which may avoid the risk of ventilatory depression induced by intravenous opioid administration. Because of the absence of side effects, such as vertigo from general anesthesia and intravenous opioids, patients receiving epidural anesthesia and analgesia tend to ambulate earlier than patients receiving general anesthesia. Early ambulation may decrease the incidence of postoperative pulmonary complication in obese patients (Rawal et al. 1984).

In conscious patients, increased minute ventilation during laparoscopic procedures may lead to increased respiratory rate (Ciofolo et al. 1990), creating difficulty in performing the procedure. Although in our cases, the experienced surgeon considered the abdominal relaxation sufficient to continue, increased respiratory rate caused a slight disturbance during exploration in case 1. The operative time was 49 min in this patient, which was favorable compared with previous surgeries by the same team. In their previous study, mean operative time was 60.63 ± 27.37 min in patients under general anesthesia (Zachariah et al. 2013). This may imply that an experienced surgeon can handle a slightly increased respiratory rate. Other studies focusing on laparoscopic cholecystectomy also demonstrated no difference in operative time between regional and general anesthesia (Bessa et al. 2012). Further studies are needed to elucidate this issue.

There are some limitation in this report. First, difficulty in anesthetic technique may be encountered more frequently in obese patients, which may lead to a high failure rate when performing epidural anesthesia. Secondly, intraoperative conversion of epidural anesthesia to general anesthesia may be required if patients cannot tolerate the laparoscopic procedure (e.g. intolerable shoulder pain or prolonged surgical time). Finally, increased respiratory rate in awake patients during pneumoperitoneum may lead to difficulty in performing laparoscopic surgery. In this condition, conversion to general anesthesia is also required. These concern may discourage anesthesiologists and surgeons to perform this anesthetic technique for laparoscopic bariatric surgery. Further prospective studies with large sample size are required to clarify the indication and contraindication (relative advantages and disadvantages) of performing regional anesthesia for laparoscopic bariatric surgery.

## Conclusion

This report reminds the anesthesiologists and surgeons that epidural anesthesia may be a viable alternative to general anesthesia for laparoscopic bariatric surgery in selected cases. This anesthetic technique may maintain pre-operative respiratory function, increase alertness, and reduce the use of rescue analgesics. A speedy return to baseline function is crucial for optimal outcomes in these patients.

## Consent

Both patients provided written consent for publication of this report.

## Abbreviations

COPD: chronic obstructive pulmonary disease
FVC: forced vital capacity
FEV1: one-second forced expiratory volume
